# Successful management of aggressive fibromatosis of the neck using wide surgical excision: a case report

**DOI:** 10.1186/1752-1947-5-244

**Published:** 2011-06-27

**Authors:** Zain A Sobani, Montasir Junaid, Mumtaz J Khan

**Affiliations:** 1 Medical College, Aga Khan University, Stadium Road, Karachi, Pakistan; 2 Division of Otolaryngology, Head & Neck Surgery, Aga Khan University, Stadium Road, Karachi Pakistan

## Abstract

**Introduction:**

Aggressive fibromatosis is a benign tumor, thought to arise from deep musculoaponeurotic structures, rarely found in the head or neck. However, when it does occur in the head and neck region, it tends to be more aggressive and associated with significant morbidity, which may be attributed to the vital vascular, neurological or anatomical structures in close proximity.

**Case presentation:**

We report the case of a 39-year-old Pakistani man who presented with a two-month history of a lump on the right side of his neck. The mass was excised and histopathological analysis revealed a case of aggressive fibromatosis.

**Conclusion:**

Due to the rarity of the condition no guidelines are available on the indications and extent of each modality. Due to its aggressive behavior and tendency to invade local structures and recur, a multi-modality management strategy is usually employed. On the basis of this case, we suggest that aggressive surgery is a viable management option and may be successfully used as a single modality treatment.

## Introduction

Aggressive fibromatosis (AF) is a benign tumor, thought to arise from deep musculoaponeurotic structures [1], characterized by local invasion and recurrence that can occur anywhere in the body. It has rarely been reported in the head and neck region, with 179 cases documented between 1968 and 2008.

Due to the tendency of AF to invade local structures and recur, a multi-modality management strategy is usually employed. However due to its rarity in the head and neck, no guidelines are available on the indications and extent of each modality. Here we report a case of AF of the neck successfully managed by wide surgical excision; with no signs of recurrence 22 months after surgery.

## Case presentation

A 39-year-old Pakistani man had presented to our General Surgery clinic with a two-month history of a lump on the right side of his neck. There were no associated symptoms. There was no history of tobacco or irritant use. On physical examination a fixed mass, 4 cm in size, was palpated in right level III. It was not associated with any changes in the overlying skin. An examination of his upper aerodigestive tract was unremarkable. The mass was excised by a general surgeon. Histopathological analysis demonstrated spindle-shaped cells with no identifiable nuclear pleomorphism or mitotic activity. The cells stained positive for anti-smooth muscle actin (ASMA), favoring a smooth muscle origin.

The mass, however, recurred within a period of one month and continued to increase in size, limiting his neck movement to the right side. On presentation to our Head and Neck Surgery clinic, a hard, well demarcated, non-tender and non-mobile mass of about 5 × 8 cm was noticed in the posterior triangle.

A computed tomography (CT) scan of his neck showed a large soft tissue mass in his right posterior neck, measuring about 6.5 × 4.7 cm in its greatest dimension, involving both his anterior and posterior scalene muscles and closely abutting the right lobe of his thyroid gland. On the anterior side it extended up to his sternocleidomastoid muscle (SCM). Splaying of his right internal jugular vein was also noted between the SCM and the mass. Multiple sub-centimeter lymph nodes were also noted at level II. Fine-needle aspiration cytology (FNAC) showed spindle shaped cells with elongated vesicular nuclei and pink cytoplasm admixed with pink fibrocollagenous material suggestive of fibromatosis. Our patient underwent a wide local excision with a right-sided neck dissection.

Histopathological analysis of the excised tissue showed spindle cells arranged in sheets and intersecting fascicles with variable collagen deposition. No cytological atypia was noted; however, infiltrative growth was noticed within the entrapped skeletal muscle at the periphery. The lesion showed evidence of vascular proliferation and peripheral lymphocytic infiltration. The above findings correlated with a tissue diagnosis of AF.

**Figure 1:**
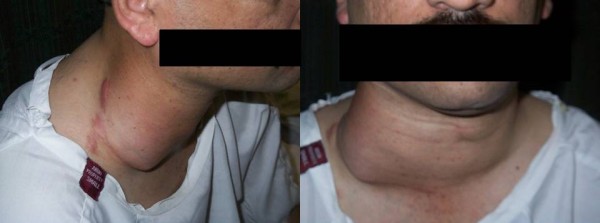
**39-year-old male with a right sided neck mass**. Scars from previous excisions are seen. The reccurring mass on examination.

Our patient once again presented with local recurrence of the mass [Figure 1], not associated with any pain or tenderness, but with complaints of suffocation when lying supine, due to a pressure effect generated by the mass [Figure 1].

**Figure 2:**
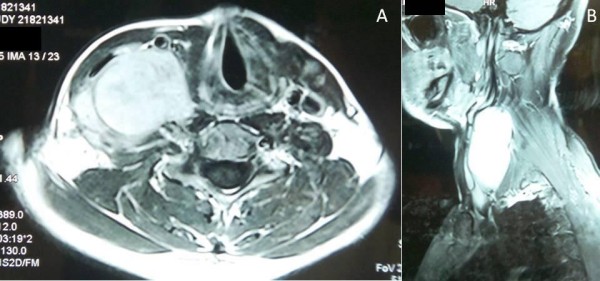
**Axial (A) and sagittal (B) T1 MRI scans with contrast showing the mass**.

**Figure 3:**
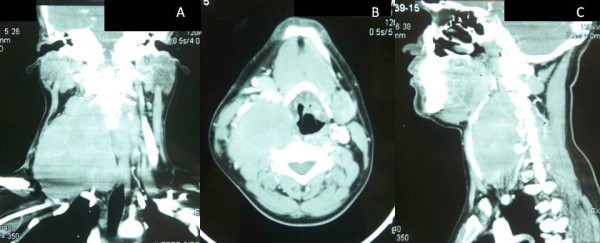
**Coronal (A), axial (B) and sagittal (C) CT scans with contrast showing the mass measuring about 9.3 × 5.9 × 12 cm with areas of central necrosis**. The carotid sheath is displaced anteriorly.

Magnetic resonance imaging (MRI) showed a large contrast-enhancing mass, hypointense on T1 and hyperintense on T2, lying in the lower part of his neck to the right of the midline. It was deep to the SCM on the anterior side, abutting the larynx medially and the paravertebral muscles and space on the posterior side [Figure 2]. A CT scan showed a large soft tissue density mass measuring about 9.3 × 5.9 × 12 cm, with central necrosis consistent with recurrent fibromatosis in the posterior triangle [Figure 3].

**Figure 4:**
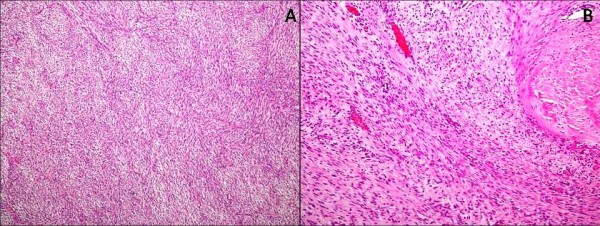
**Histopathological examination of the excised tissue**. (A) Fibrosarcoma arising in a background of dermatofibrosarcoma protuberans showing proliferation of spindle cells with a prominent storiform pattern and cells exhibiting pleomorphism with increase mitotic activity (H&E, 10 ×). (B) Fibromatosis showing proliferation of bland spindle shaped cells with infiltration into the muscle at the periphery and perivascular lymphocytic infiltrate (H&E, 10 ×).

The mass was resected and an extended right neck dissection was performed. Analysis of the excised tissue revealed spindle cells arranged in whorls and fascicles, with intervening thin walled vessels and extravasation of red blood cells. There was no evidence of atypical mitosis or necrosis; however, focal infiltration of adjacent muscle was noted, favoring benign fibromatosis [Figure 4].

Our patient was followed in the out-patient clinic regularly for 22 months with no evidence of recurrence of disease. No further treatment was given.

## Discussion

AF in the head and neck is a rare benign tumor, thought to arise from connective tissue, fascial sheaths, and other musculoaponeurotic structures of the body [1]. It presents as a progressively enlarging painless lump, fixed to the underlying structures. By virtue of its location it may be associated with trismus, airway obstruction, dysphagia, and proptosis [2]. Intermittent airway obstruction due to pressure effects and gravitational position may also occur, as was noted in our case.

AF is a slow growing tumor; as such it demonstrates a low level of mitosis and cellular atypia, and distant metastasis has yet to be reported. Its true pathogenic potential lies in its ability to locally invade surrounding structures. This fact is of extreme importance in the head and neck region due to the compact anatomical schematics and close association with vital neurovascular and anatomical structures; however invasion of the neurovascular structures has not been reported [3].

Kruse *et al*. proposed a classification for AF in the head and neck after reviewing cases reported in the last 40 years, based on the site (such as upper and lower compartment), bone involvement and presence of hormone receptors [3]. In their review they found no indicators for potential recurrence in relation to age, sex, or localization [3], but the depth of invasion was shown to significantly affect disease-free survival [4].

The main treatment modality is wide surgical excision of the lesion, however in the head and neck region preservation of vital structures and their function may impede this objective [5]. Therefore a multi-modality management strategy is usually employed to control residual disease. Surgery is usually combined with radiation therapy for control of residual disease and to prevent recurrence.

Studies have shown that radiation therapy can also be effectively employed independently, leading to a complete response in 20%, a partial response in 20%, and stable disease in 53% of cases; supporting evidence that radiation therapy may decrease indications for surgery [6]. In cases of patients undergoing multi-modality treatment, including surgical resection and radiation therapy, the recurrence free survival rate is 83.6% at five years [6], with figures going up to 88.5% for 10-year recurrence free survival [7].

However, radiation therapy is associated with a high risk of complications, especially in the head and neck region, and in our opinion should only be considered in cases with residual disease or where surgery may significantly impair the functional capabilities of the patient.

Although it is argued that chemotherapy is not effective in benign tumors with low mitotic rates, cases of AF successfully treated with cytotoxic and non-cytotoxic chemotherapy are being reported. Sze *et al*. report significant tumor shrinkage with low dose methotrexate and vinblastine [1]. Another trial concluded that pegylated liposomal doxorubicin leads to tumor shrinkage and partial remission in some cases; or at least stops proliferation, leading to a stable disease state [8]. Meloxicam, a COX-2 inhibitor, has also been shown to be effective in controlling extra-abdominal AF [9]. Keeping these in mind, pharmacological therapy can be considered for unresectable disease, or in cases where surgical and radiation therapy may lead to significant morbidity. Janis *et al*., in their review on pharmacological management of AF, concluded that although chemotherapy is effective against AF, larger trials are required to validate their results [10]. Sarcoma Alliance for Research through Collaboration (SARC) initiated a multi-center phase II trial on the efficacy of imatinib in AF and estimated progression-free survival was 94% and 88%, for two and four months respectively; whereas one year progression-free survival was 66% [11].

Recently radio frequency ablation has also been advocated by Ilaslan *et al*., with no recurrence noted after a mean follow-up of 30 months [12]. Early data from the Mayo Clinic, USA shows that percutaneous cryoablation may provide an alternative treatment for small to middle sized tumors [13]. However, both modalities have been recently tried on a limited number of patients and it is too early to comment on their application.

## Conclusion

The rarity of AF in the head and neck means that no guidelines are currently available on the indications and extent of each modality. Due to the aggressive behavior and tendency to invade local structures and recur, a multi-modality management strategy is usually employed. On the basis of this case, the authors suggest that aggressive surgery is a viable management option for AF in the head and neck, and may be successfully used as a single modality treatment. However where functional preservation and aesthetics need to be taken into account, a multi-modality treatment plan involving chemotherapy and radiation therapy can be considered for its management.

## Consent

Written informed consent was obtained from the patient for publication of this case report and any accompanying images. A copy of the written consent is available for review by the Editor-in-Chief of this journal.

## Competing interests

The authors declare that they have no competing interests.

## Authors' contributions

ZAS and MJ were primarily involved in compiling patient details, reviewing available literature and drafting the primary manuscript with contributions from the other authors. MJK identified the patient and interpreted the patient data along with contributing to and editing the manuscript. All authors read and approved the final manuscript.
